# Marjolin cutaneous squamous cell carcinoma arising in dissecting cellulitis of the scalp

**DOI:** 10.1016/j.jdcr.2024.07.020

**Published:** 2024-08-12

**Authors:** Abena Maranga, Advaitaa Ravipati, Maria Estela Martinez-Escala, Christopher R. Shea, Amy Z. Xu

**Affiliations:** a Section of Dermatology, Department of Medicine, University of Chicago Medicine, Chicago, Illinois; b Dr. Phillip Frost Department of Dermatology and Cutaneous Surgery, University of Miami Miller School of Medicine, Miami, Florida

**Keywords:** cutaneous squamous cell carcinoma, dissecting cellulitis, follicular occlusive disorder, Marjolin ulcer, scarring alopecia

## Introduction

Dissecting cellulitis of the scalp (DCS) is a chronic inflammatory disorder characterized by sterile abscesses and tender, suppurative nodules resulting in interconnected sinus tracts and end-stage keloidal scarring. It may occur alone or in the context of other follicular occlusive disorders, including acne conglobata, hidradenitis suppurativa (HS), and pilonidal cysts. Cutaneous squamous cell carcinoma (cSCC) arising within long-standing inflammatory lesions, also known as Marjolin ulcer, is a rare yet well-documented complication of follicular occlusive disorders, particularly in the setting of HS; an aggressive course, high postsurgical recurrence rate, and delays in diagnosis are common.^1^ Here, we present a case of Marjolin cSCC arising within DCS, a phenomenon reported once previously in the literature, to emphasize a rare and diagnostically challenging complication of long-standing disease.

## Case

A 66-year-old man with a history of anemia and intermittent alcohol abuse presented to dermatology clinic with an enlarging, tender scalp mass. He noted persistent purulent and occasionally bloody drainage for months, and sharp pains that interrupted sleep. Prior history was notable for a nearly 30-year history of biopsy-proven DCS involving the area of concern, treated with multiple modalities including topical steroids, intralesional steroid injections, and oral antibiotics. A recurrence of tenderness, odor, and drainage had prompted him to return to clinic after a prolonged period of stability.

**Fig 1:**
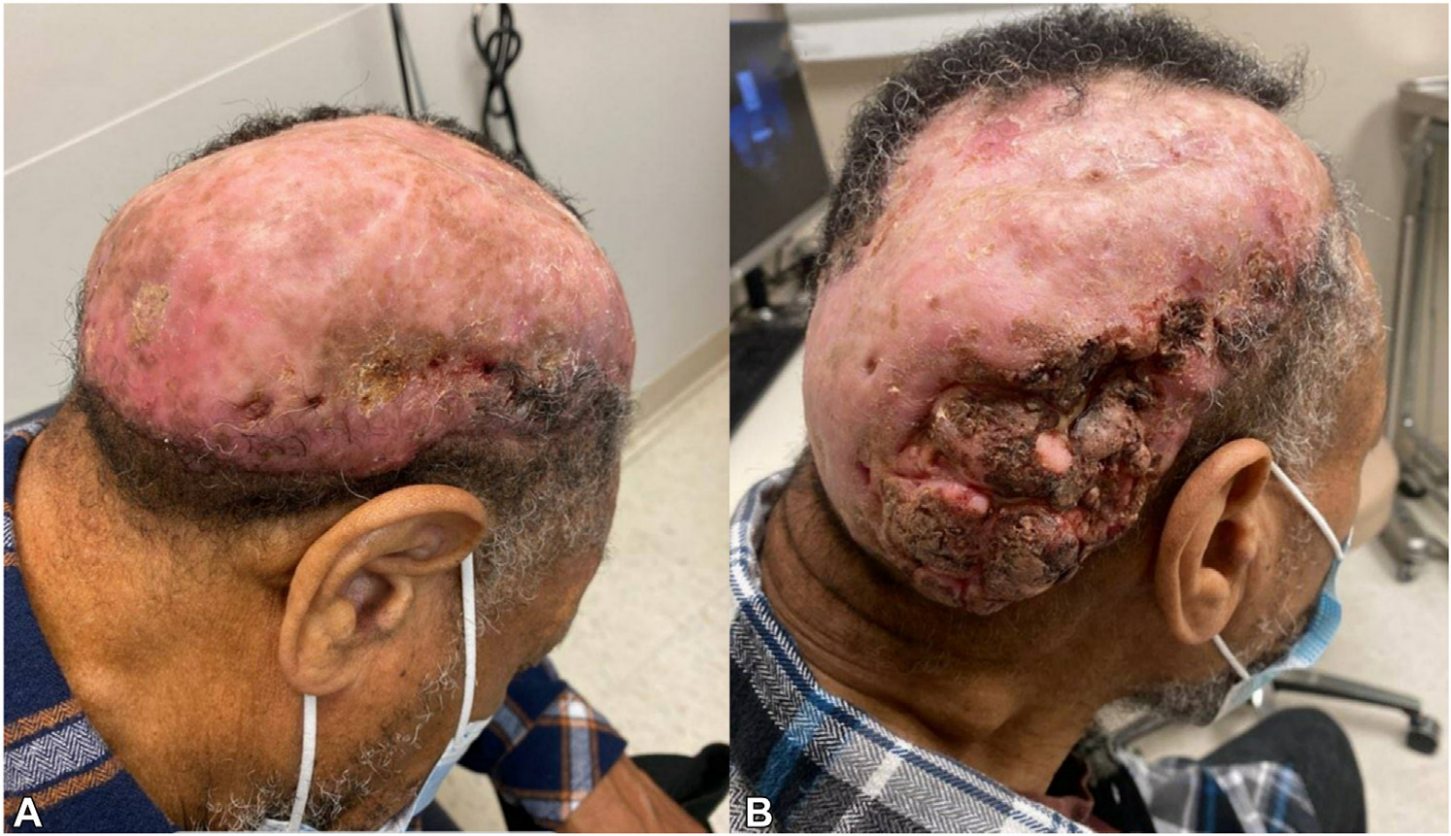
**A,** Long-standing dissecting cellulitis of the right parietal-occipital scalp with keloidal scarring and alopecia, and associated reactive right-sided lymphadenopathy. **B,** Marked interval development of an exophytic cribriform mass at the time of biopsy confirming squamous cell carcinoma.

Clinical examination showed a large, sclerotic, alopecic, oblong mass with complete follicular obliteration extending from the occipital scalp to the right frontal scalp, and a focal nodule at the right parietal scalp with cribriform surface and purulent drainage (Fig 1, *A*). Numerous enlarged lymph nodes on the right cervical chain were palpable. Given the suspicion for a recurrence of his underlying dissecting cellulitis with overlying superinfection, a tapering course of oral prednisone 40 mg daily combined with doxycycline was begun. A 14-day course of cephalexin was added after bacterial cultures grew *Streptococcus agalactiae*. Due to persistent activity after 6 weeks, his treatment was changed to adalimumab.

**Fig 2:**
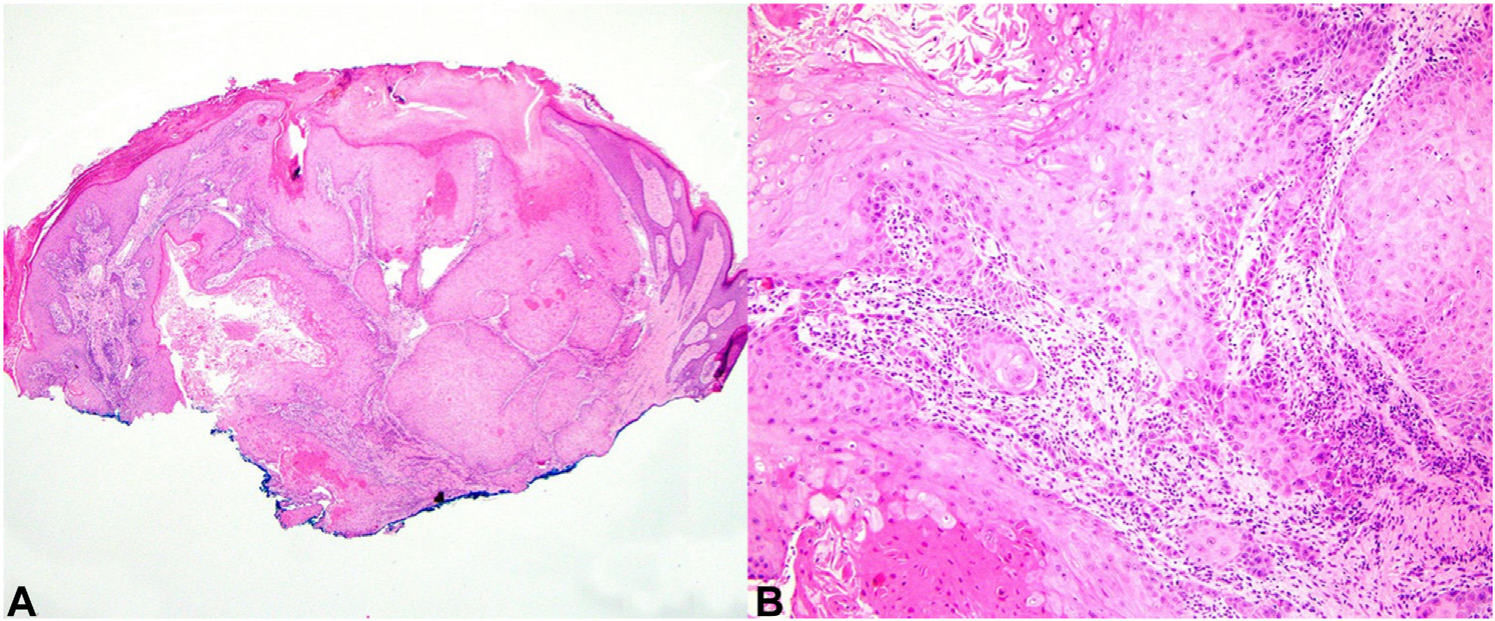
**A,** Histopathology showing well-differentiated invasive squamous cell carcinoma. **B,** Small tumor islands and cords of atypical keratinocytes are present in the dermis. Hematoxylin-eosin stain; original magnifications: (**A**) 2×, (**B**) 20×.

At 2-week follow-up, the right parietal nodule had rapidly progressed into an exophytic, fungating tumoral mass (Fig 1, *B*). All immunosuppressants were withheld. Punch biopsy of the right occipital mass confirmed well-differentiated invasive cSCC (Fig 2, *A* and *B*).

The patient was referred urgently to Oncology. Head and neck computed tomography (CT) scan demonstrated a 14 × 10 × 5 cm tumor of the right parietal-occipital scalp; positron emission tomography–CT scan showed multiple hypermetabolic lymph nodes in the bilateral neck, right axilla, and chest wall. No distinct visceral metastases were noted. He was started on pembrolizumab 200 mg every 3 weeks; carboplatin, paclitaxel, and radiation therapy were subsequently added due to progression of disease. The patient ultimately underwent wide local excision with latissimus dorsi free-flap reconstruction approximately 1 year after initial cSCC diagnosis; all surgical margins and lymph node dissections were negative for residual disease. He continues to follow with Oncology and Otolaryngology for surveillance CT scans every 6 months.

## Discussion

Cutaneous SCCs arising within long-standing lesions of follicular occlusive disorders are known to be highly aggressive. Recurrent cycles of tissue destruction and re-epithelialization, persistent hyperproliferative wound healing responses, and extensive scarring resulting in an immunologically privileged site are factors thought to contribute to the aggressiveness of such tumors.^2^^,^^3^ In a review of 43 cases of HS complicated by cSCC, 36% exhibited lymph node or distant metastasis, and nearly half developed recurrence after surgical excision, with mortality rate approaching 50%.^4^ Delays in diagnosis are unfortunately common, likely due to difficulty in distinguishing recrudescence of inflammation from malignant transformation, as well as from high false-negative biopsy rates secondary to the depth and focality of carcinomatous changes.^1^ Multiple tissue biopsies from various locations may be required to capture foci of frank cSCC.^5^^,^^6^

Although cSCC arising within HS is documented in more than 80 case reports, only 1 case of cSCC developing within DCS has been published previously.^6^^,^^7^ Similar to our case, the patient presented with acute drainage and pain of long-standing DCS of the occipital scalp; notably, the scalp lesions did not appear significantly changed from baseline several months prior. He was started on oral dapsone for presumed DCS flare, but returned 2 months later with interval development of a large, fungating mass. Several biopsies were performed before the diagnosis of cSCC was made, and despite undergoing immediate wide local excision with negative margins, he developed numerous tumoral metastases and expired less than 4 months later.

We present this case to highlight a rare and potentially highly morbid phenomenon occurring as a late complication of DCS. The initial recurrence of our patient’s symptoms after a prolonged period of disease quiescence likely represented malignant degeneration into cSCC, with tumor growth accelerating after the addition of adalimumab. As exemplified by our case and that of Curry et al, active inflammatory DCS manifesting as fluctuant, tender, and draining nodules may be difficult to distinguish from malignant transformation. Thus, early and frequent biopsy remains crucial for excluding the development of cSCC within long-standing lesions, and a high index of suspicion is required for patients with sudden recrudescence of inflammatory symptoms after prolonged stable disease.

## Conflicts of interest

None disclosed.
